# Mapping the visual brain areas susceptible to phosphene induction through brain stimulation

**DOI:** 10.1007/s00221-016-4784-4

**Published:** 2016-09-28

**Authors:** Lukas F. Schaeffner, Andrew E. Welchman

**Affiliations:** Department of Psychology, University of Cambridge, Downing Street, Cambridge, CB2 3EB UK

**Keywords:** Transcranial magnetic stimulation, Brain stimulation marker, Cortical excitability, Stimulation efficacy

## Abstract

Transcranial magnetic stimulation (TMS) is a non-invasive brain stimulation technique whose effects on neural activity can be uncertain. Within the visual cortex, phosphenes are a useful marker of TMS: They indicate the induction of neural activation that propagates and creates a conscious percept. However, we currently do not know how susceptible different areas of the visual cortex are to TMS-induced phosphenes. In this study, we systematically map out locations in the visual cortex where stimulation triggered phosphenes. We relate this to the retinotopic organization and the location of object- and motion-selective areas, identified by functional magnetic resonance imaging (fMRI) measurements. Our results show that TMS can reliably induce phosphenes in early (V1, V2d, and V2v) and dorsal (V3d and V3a) visual areas close to the interhemispheric cleft. However, phosphenes are less likely in more lateral locations (hMT+/V5 and LOC). This suggests that early and dorsal visual areas are particularly amenable to TMS and that TMS can be used to probe the functional role of these areas.

## Introduction

Transcranial magnetic stimulation (TMS) is a non-invasive technique that can be used to temporarily disrupt normal neural activity (Robertson et al. 2003; Walsh and Pascual-Leone 2003; Sandrini et al. 2011). This makes it possible to investigate the causal relationship between particular cognitive functions and the network of brain activity that supports those functions (Pascual-Leone et al. 2000; de Graaf and Sack 2014).

However, the efficacy of TMS-related effects relies on a great number of parameters, for instance the timing, intensity, duration, or current flow direction of stimulation (Robertson et al. 2003; de Graaf and Sack 2011; Sandrini et al. 2011). This poses an interpretative challenge to experimenters: When we apply TMS, we need some reassurance that the method can effectively change neural activity at a particular target site in the brain (de Graaf and Sack 2011).

In most regions of the brain, it is difficult to directly observe the effects of TMS since there is no immediate, overt perceptual or behavioural response. However, in limited areas of the brain, TMS triggers a response making it possible to probe the efficacy of TMS at the target location. In particular, in the visual cortex, TMS can result in an visual phosphene (Marg and Rudiak 1994) that provides a measure of whether a given stimulation protocol evokes sufficient neural excitation to reach conscious awareness (Walsh and Pascual-Leone 2003; de Graaf and Sack 2011; Silvanto 2013). Thus, this marker is useful in identifying that a particular portion of the cortex is amenable to testing using TMS.

 Nevertheless, there is some uncertainty about exactly which parts of the visual cortex will yield a phosphene through stimulation. Previous work reported that phosphenes are induced most reliably over early visual cortex near the cortical midline (Marg and Rudiak 1994; Kammer et al. 2005), although this work did not investigate the stimulation outcome for all identified retinotopic visual areas.

Other work has suggested stimulation locations relative to anatomical landmarks such as the inion (Gerwig et al. 2003; Elkin-Frankston et al. 2010). However, these suggestions vary between studies, and there is evidence that functional brain architecture is not well described by scalp landmarks (Sack et al. 2009).

Salminen-Vaparanta et al. (2014) used detailed retinotopic maps and current modelling to show that separate stimulation of both V1 and V2d is equally capable of inducing phosphenes. Their approach underlines that we need to have knowledge of the individual functional structure of the visual cortex if we want to understand where in the brain phosphenes can be induced.

Here we therefore sought to assess the efficacy of TMS for phosphene induction where we had an understanding of which portions of visually responsive cortex were being targeted by TMS. In particular, we systematically map out the locations at which participants report phosphenes and relate these to the retinotopic organization and the location of object- and motion-selective areas of the visual cortex as revealed by functional magnetic resonance imaging (fMRI) measurements. To anticipate, our results demonstrate that phosphenes are induced reliably over early visual areas (V1, V2d, V2v) and dorsal areas (V3d, V3a), and confirm previous observations that phosphenes are more likely to be induced close to the cortical midline (Marg and Rudiak 1994; Kammer et al. 2005).

## Methods

### Participants

We tested 30 healthy participants (18 females; age range from 20 to 38, *M* = 26.43, SD = 4.32, including the author L.F.S.) to determine whether they perceived phosphenes under TMS stimulation (see “Phosphene screening” section). Before the experiment, participants provided written informed consent and were screened for contraindications to fMRI and TMS (Wassermann 1998; Rossi et al. 2009). Procedures were approved by the University of Cambridge ethics committee and were performed in accordance with the ethical standards laid down in the Declaration of Helsinki. Twenty-one participants (70 %) reported a percept after stimulation, the remaining nine did not report experiencing a phosphene under our TMS protocol. Eight participants reported a percept after control stimulation and were therefore excluded from the experiment. One participant aborted the screening procedure complaining of the side effects of TMS. Twelve participants reported phosphenes reliably. Of these, seven participants (two females; age range from 23 to 32, *M* = 26.29, SD = 3.4, including the author L.F.S.) agreed to continue to the main experiment.

### Experimental set-up and stimulation

The experiment was conducted in a dimly lit room using a black screen with low luminous intensity (0.15 cd/m^2^). We instructed participants to maintain fixation at a bright dot in the centre of the screen. We allowed 5 min for adaptation to the illumination before the start of the experiment.

We applied single TMS pulses with a biphasic MagStim Rapid^2^ stimulator (MagStim, Whitland, UK) via a figure-of-eight coil (outer winding diameter = 70 mm). Throughout the experiment (“Phosphene Screening”, “Cortical excitability”, “Mapping phosphenes”, “Phosphene induction with different current directions”, and “Phosphene induction with different stimulation protocols” sections), a minimum stimulation onset asynchrony of 3 s was used to avoid TMS-related long-term effects and muscle fatigue (Kammer et al. 2001a, 2005). The induced current direction (during the initial, rising phase of the biphasic waveform) was lateral to medial in the targeted hemisphere (Kammer et al. 2007; Taylor et al. 2010) and the coil handle pointed away from the head laterally. Stimulation was applied over the left hemisphere.

### Phosphene screening

Phosphenes were described to participants as flashes of light or distortions of the visual field. We provided verbal and graphic illustrations of phosphenes described by previous literature (Marg and Rudiak 1994). Participants were asked to give a conservative yes–no response, only reporting a percept when they were absolutely sure. At the start of the experiment, participants performed a control task to test whether phosphenes could be induced and to validate the percept:

(i) Feedback about the percept had to match previous descriptions from Marg and Rudiak (1994).
(ii) Phosphenes had to appear in the visual hemifield contralateral to the stimulated hemisphere or both hemifields, due to the organization of the early visual cortex (Kammer et al. 2005).
(iii) Perception of phosphenes had to be possible with eyes open and closed (Kammer and Baumann 2010; Fried et al. 2011).
(iv) Stimulation of brain tissue distant from the visual cortex, over the vertex (Cz), should not produce a percept (Fried et al. 2011).

Participants were tested for phosphenes with a hunting procedure in a 4 × 4 cm window over the visual cortex. The centre of the window was located 4 cm caudal and 2 cm lateral relative to the inion (Gerwig et al. 2003; Elkin-Frankston et al. 2010). For two participants, an MRI anatomical scan was available prior to the experiment. For these participants, hunting stimulations were applied at 16 equally spaced stimulation targets. For the remaining participants, stimulations were applied randomly within the defined window on the scalp. We applied stimulation at 80 % stimulator output, approximately 130 % of the average phosphene threshold reported in previous studies using the same stimulator and coil model (Abrahamyan et al. 2011; Stokes et al. 2013).

We applied 48 hunting stimulations. The coil was moved to a new location after each hunting stimulation. If participants reported a phosphene, ten stimulations with eyes open and ten stimulations with eyes closed were applied at the same location to assess how frequently phosphenes could be induced. The screening was successfully finished after participants described phosphenes at five different stimulation locations. If participants did not describe a percept in 48 hunting stimulations, the screening was aborted. At three different times during the screening, ten control stimulations were applied over the vertex (Cz). The number of stimulations we applied during screening depended on the performance of the participant and could range from 48 stimulations (no phosphenes perceived) to 178 stimulations. Participants who reported >1 phosphene after vertex stimulation (*n* = 8) or could not perceive more than three phosphenes out of ten TMS pulses at any stimulation location (*n* = 9) were excluded from the experiment.

### Functional magnetic resonance imaging

Data were acquired with a three-tesla scanner. For all participants, a high-resolution anatomical scan (1 mm^3^) was acquired. For all scans, blood oxygen-level-dependent signals were measured with an echo-planar imaging sequence. Retinotopic areas V1, V2d, V3d, V3a, V2v, V3v, and V4 were defined with standard retinotopic mapping procedures using rotating wedge stimuli. The borders between functional areas were defined by the resulting angular maps (Wandell et al. 2007). We identified the hMT+/V5 complex as a group of voxels that responded significantly more (*p* < 0.01) to a coherently moving array of dots than to a static array of dots (Zeki et al. 1991). The lateral occipital complex (LOC) was mapped as the set of voxels that responded significantly (*p* < 0.01) stronger to intact than scrambled images of objects (Kourtzi et al. 2005). We analysed fMRI data with BrainVoyager QX (BrainInnovation B.V.).

### Neuronavigation

We created a curvilinear reconstruction of the cortex from anatomical MRI data. We used a fully automated algorithm provided by Brainsight 2.2.12 (Rogue Research, Montreal, Canada) which is based on the Brain Extraction Tool (Smith 2002). We “peeled” the reconstruction 4 mm deep to guarantee that stimulation targets were located within the cortex. The curvilinear reconstruction was co-registered to the participant through anatomical landmarks on the head (the tip of the nose, the bridge of the nose, and the notch above the tragus for the left and right ear). During the experiment, we monitored the position of the TMS coil and the participant’s head with an infrared camera and Brainsight 2.2.12 neuro navigation software. A normal vector originating in the centre of the figure-of-eight TMS coil helped to guide the coil to a defined location over the curvilinear reconstruction.

**Fig. 1:**
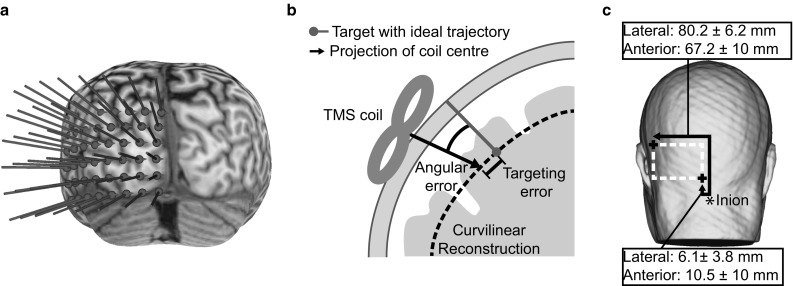
**a** Posterior view of the curvilinear reconstruction of the cortex (peel depth 4 mm) for one participant. Stimulation targets were placed equidistant over the visual cortex with a 5 mm offset to the interhemispheric cleft and the cerebellum. **b** Illustration of online coil monitoring during the experiment with stereotactic neuronavigation. One stimulation target and the ideal trajectory are shown in red on the curvilinear reconstruction (*dashed line*). The trajectory marks the ideal coil position on the scalp. A vector projecting from the centre of the TMS coil defines the current position relative to brain and stimulation target. For the displayed coil position, the targeting error (distance on the curvilinear surface) and the angular error (angle of the coil vector with respect to the target trajectory) are shown. **c** Window of coil locations on the scalp (*dashed line*) for stimulation targets in **a**. The outer borders of an average stimulation window for all participants are described as the offset (Mean ± SEM in mm Euclidean distance) of two corner points (*plus sign*) to the inion (*asterisk*) along the scalp

We generated a 6 × 8 stimulation target grid (10 mm inter-target distance). We placed this over the curvilinear cortical reconstruction with a 5 mm offset from the interhemispheric cleft and the cerebellum (See Fig. 1a). For each target, an ideal trajectory was defined approximately normal to the curvilinear surface. A targeting error was defined as the distance from a target in the brain to the vector projecting from the coil into the human head. Angular error was defined as the angle of the coil vector with respect to the target trajectory (See Fig. 1b). During stimulation, both values were monitored, targeting error was kept <1 mm, angular error was kept <15° as suggested by the Brainsight 2.2.12 manual.

The coil positions for respective targets in the brain covered a large window on the scalp at the back of the head. The location of this window is described relative to the inion for easy replication without stereotactic neuronavigation (See Fig. 1c).

### Cortical excitability

For each participant, we defined an excitation threshold, using the REPT adaptive staircase method (Abrahamyan et al. 2011). REPT estimates excitation thresholds from 30 stimulations. Thresholds mark the stimulation intensity (% stimulator output) at which a phosphene can be elicited in 50 % of the stimulations.

We defined the phosphene threshold at the grid target closest to the centre of area V3d (This target tended to lie in the centre of the window that was used in the initial phosphene screening test). Stimulations were applied in a range of 45–90 % stimulator output. For the remaining 47 stimulation targets, we used an adjusted phosphene threshold calculated by correcting stimulation intensity for the distance to the underlying cortical surface (Stokes et al. 2013). We obtained the surface of the cortex with the segmentation routine (Kriegeskorte and Goebel 2001) from Brainvoyager QX 2.8 (Brain Innovation, Maastricht, the Netherlands). We then calculated the average distance from the TMS coil on the scalp to the closest 100 vertices of the cortical surface segmentation (Cai et al. 2012). For all targets, this method gave a slightly closer distance estimation than the estimate used by Stokes et al. (2013) (mean difference −2.17 mm, SD 0.52 mm).

### Mapping phosphenes

**Fig. 2:**
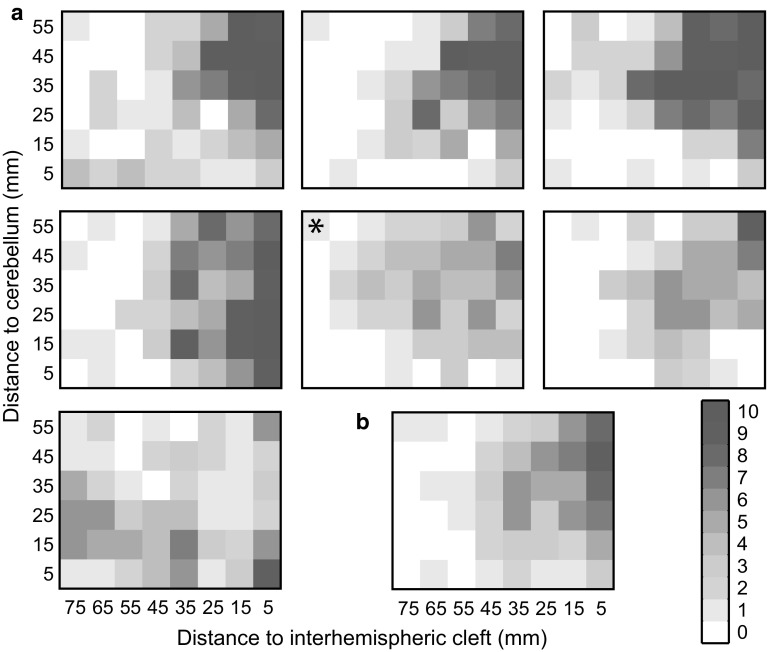
Individual (**a**) and median (**b**) stimulation results for all participants (*n* = 7). The colour of the heatmap indicates the number of phosphenes that were perceived in ten stimulations at the defined targets shown in Fig. 1a. An *asterisk* marks the stimulation results for one participant for whom stimulation was always applied at the maximum stimulator output used in this experiment (colour figure online)

In the main experiment, stimulation at each grid target was set at 110 % of the estimated phosphene threshold (Salminen-Vaparanta et al. 2014). For some targets, stimulation intensity suggested by the adjustment algorithm exceeded 90 % stimulator output which we used as an upper limit on stimulation intensity. In these cases, stimulation was delivered at 90 % of the stimulator’s output. For one participant with a high phosphene threshold, all targets reached this correction limit; hence, no correction was performed. Stimulation results for this participant are marked in Fig. 2a. In another four participants between 1 and 4 targets were corrected. Overall, for 59 out of 336 targets, the stimulation intensity was corrected to an upper limit.

An important question is whether this correction limit systematically affected the stimulation outcome. Targets where stimulation intensity was corrected to an upper limit were located in almost all functional areas [V1(×1), V2v(×1), V2d(×6), V3d(×4), V3a(×5), hMT+/V5(×2), LOC(×8)]. Of the corrected stimulations (*n* = 590), 23 % induced a phosphene. For comparison, considering all stimulations applied in this study, 30 % induced a phosphene. It is therefore possible that the outcome of stimulations, where intensity was capped at an upper limit, underestimates the susceptibility of the targeted areas for phosphene induction. However, this correction limit was applied mostly in both areas with a high (V3, V3a) and a low (hMT+/V5, LOC) phosphene incidence which shows that this did not uniquely affect specific areas.

The intensities used in this experiment ranged from 48 to 90 % stimulator output. For each grid location, 10 stimulations were given, totalling 480 stimulations per participant. The order of the stimulations for all targets was randomized. Participants were tested on separate days in three sessions that lasted approximately two hours (160 stimulations per session).

### Defining the location of TMS effects in the brain

We located the centre of gravity (CoG) of TMS which estimates the point on the cortical surface where the maximum electric field is induced. For this we used a balloon inflation projection method (Okamoto and Dan 2005). The algorithm uses the centre position of the coil on the scalp and a segmentation of the grey matter surface (~140,000 vertices for one hemisphere). We identified the 200 surface points closest to the coil centre. A vector was drawn from the coil centre through the mean coordinates of the 200 surface points. We defined a rod with a 5 mm radius around the vector, given that stimulation targets in the brain were placed in 10 mm equidistant steps. The surface point within the radius closest to the vector was defined as the CoG for TMS stimulation for the given coil position. This CoG was used to assign stimulation effects to underlying functional areas in the visual cortex.

This algorithm takes into account the local curvature of the cortex and gives a more realistic estimate of the location of strongest current induction than a perpendicular vector projection from coil to cortical surface (Diekhoff et al. 2011; Weiss et al. 2013). Specifically, this projection method is not affected by coil tilt, whereas perpendicular projections have been found to overestimate the effect of coil tilt for a range of up to 15° used in this study (Opitz et al. 2013).

To validate the CoG locations from our projection method, we created a realistic current model for stimulation at 17 coil positions that targeted functional areas in one participant. We used simNIBS 2.0 (www.simnibs.org; Windhoff et al. 2013) to model current distributions with a finite element method. This model respects the effects of coil tilt as well as the effects of different tissue conductivity and individual cortical architecture on the induced current. We constructed a finite element method model consisting of 1.1 million tetrahedra based on a structural MRI. We assigned electrical conductivities to different tissue types as described by Windhoff et al. (2013). Isotropic conductivity was assumed. A magnetic dipole model for a MagStim 70-mm figure-of-eight coil was provided by simNIBS. We simulated stimulation for all targeted areas with coil position coordinates as used in the experiment. Stimulator output for a given stimulation intensity was defined relative to the peak current at 100 % stimulator output as provided by MagStim. Since the output is a sinusoidal waveform, stimulator output was calculated as the root mean square of the peak current for a pulse duration of 300 ms.

To validate that functional areas received stimulation as predicted by our projection method, we defined an area of stimulation for each coil position based on the current model: This area was defined as all surface points where the electric field intensity was between 80 and 100 % of the maximum current (Wagner et al. 2009). By comparing which of these surface points fell into which functional areas, we defined the functional area that received the maximum amount of stimulation as the target for a given coil position.

### Phosphene induction with different current directions

We retested three participants in a control study to test whether current direction of TMS systematically affected the stimulation outcome. Participants received 20 training stimulations with the stimulation parameters of the main experiment (“Phosphene mapping” section) to confirm that TMS still created a percept. Additionally, all participants received ten control stimulations over Cz to reconfirm that percepts were not induced through stimulation side effects.

We applied stimulations with the original lateral-to-medial current direction as well as three alternative current directions: posterior to anterior (coil rotated 90° counterclockwise), medial to lateral (coil rotated 180° counterclockwise), and anterior to posterior (coil rotated 270° counterclockwise). A range of 360° was tested because Kammer et al. (2007) found different stimulation outcomes for opposing current directions with a biphasic stimulator.

For each participant, we applied stimulations for all current directions at nine coil locations: three locations that yielded no phosphenes during phosphene mapping, three locations that yielded a small number of phosphenes (1–5 out of 10 stimulations), and three locations where a high number of phosphenes was induced (6–10 out of 10 stimulations). This allowed us to assess whether current orientation systematically affected the differences in susceptibility to phosphene induction that we observed during phosphene mapping.

### Phosphene induction with different stimulation protocols

In this study, 18 out of 30 participants did not perceive phosphenes reliably through TMS. It is possible that our single pulse protocol induced insufficient neural activation in these participants. We retested six participants who reported no percept through single pulse stimulation with a more powerful repetitive TMS (rTMS) protocol that was reported to induce a percept in every participant (Ray et al. 1998; Boroojerdi et al. 2002). Thirty-two pulse trains (10 Hz, 5 pulses, 0.5 s) were applied with 70 % stimulator output at original screening locations until a percept was reported. rTMS was followed by another screening (see “Phosphene mapping” section) with single pulse stimulation.

### Data analysis

We conducted statistical analysis using SPSS (IBM, Inc). We fitted a binary logistic regression model to a pooled data set of all stimulations to test whether distance of stimulation site to the interhemispheric cleft could predict the outcome of stimulation. Stimulation intensity and the distance between coil and cortical surface were included as potential covariates. Stimulations were grouped in four groups of stimulation intensity (48–60, 61–70, 71–80, and 81–90 % stimulator output) and four cortical distance groups (8–11, 12–14, 15–17, and 18–22 mm distance to the cortical surface). Since we adjusted stimulation intensity for the underlying cortical distance, the two potential covariates were correlated (*r* = .33, *p* < .01) and were therefore added in separate models. We performed polynomial contrasts to test whether there was an increase in the number of phosphenes for higher stimulation intensity or decrease for higher cortical distance. For all significant predictors, a partial correlation was derived from the respective Wald statistic.

We assessed whether the probability of inducing a phosphene changed during a two hours testing session. In particular, we compared the number of phosphenes reported over intervals of 20 stimulations (~15 min of testing) using a repeated measures ANOVA. We also calculated Cronbach’s α as a measure of test–retest reliability for the number of phosphenes reported over session intervals. Additionally, we examined whether the probability of inducing a phosphene changed between different testing sessions. We calculated Cronbach’s α for the number of phosphenes reported at different testing days.

## Results

Participants were initially screened to determine whether TMS of the visual cortex would yield the perception of phosphenes. Of the 30 people tested, we found that 12 reliably reported phosphenes. From this group, seven participants were willing to take part in the phosphene mapping experiment. We systematically applied TMS over a grid of locations covering the visual cortex (see Fig. 1a) and recorded the probability of inducing a phosphene at each location.

### Phosphene frequency at stimulation targets

Across all the TMS stimulation locations tested, we found that phosphenes were induced with a probability of 30 % (SD 6 %). Figure 2a shows a map for each participant with the number of phosphenes that were induced with ten stimulations at targets over the visual cortex. For most participants, stimulations in the dorsal visual cortex close to the interhemispheric cleft were most likely to induce a phosphene percept. Figure 2b shows this trend in a group map.

**Fig. 3:**
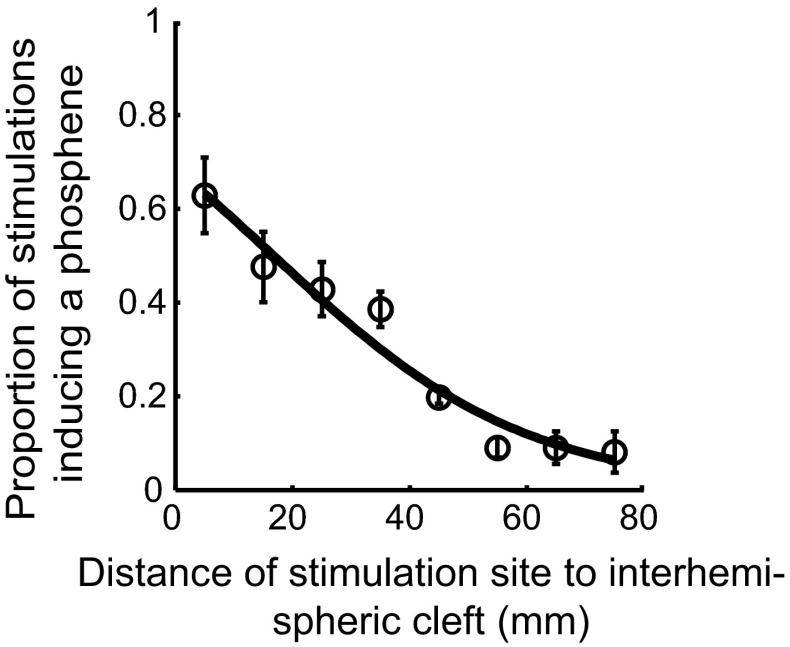
Mean proportion of TMS stimulations that induced a phosphene (*circles*) displayed as a function of the distance from stimulation site to the interhemispheric cleft. *Error bars* show the variability (SEM) of results for individual participants. A *solid line* shows the outcome of stimulation predicted for different stimulation locations by a binary logistic regression model that was fitted to the observed data

We used a logistic regression analysis to identify stimulation parameters that predict the outcome of stimulation. We found that moving the stimulation site away from the interhemispheric cleft in 10 mm steps reduced the probability of inducing a phosphene through stimulation (*B* = −0.046, SE = 0.002, *p* < .001, *R* = −.35), model fit: *χ* ^2^(1) = 624.21, *p* < .001 (Fig. 3). There was no significant increase in the number of perceived phosphenes with increasing stimulation intensity (*B* = −0.18, SE = −0.2, *p* = .370) or decrease in the number of phosphenes with larger distances to the cortical surface (*B* = −0.08, SE = 0.2, *p* = .68).

### Phosphene induction in different functional areas of the visual cortex

**Fig. 4:**
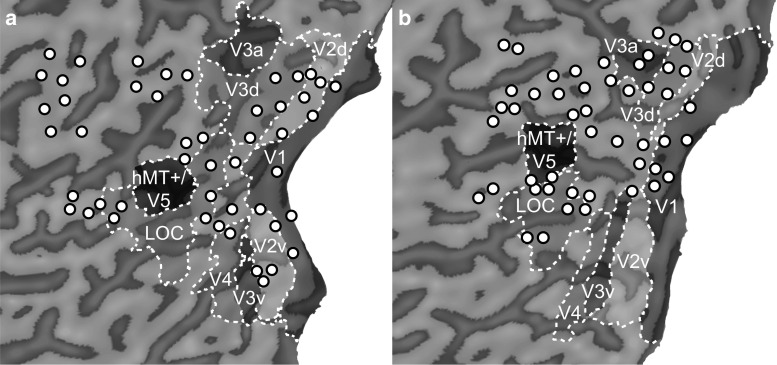
Flatmaps for two participants showing retinotopic areas, hMT+/V5 and LOC projected on the pial surface with Brainvoyager “VOI to POI plugin”. Sulci are shown in *dark grey*, gyri in *light grey*. For each stimulation target, we defined a centre of TMS-related effects (*circles*). Due to the decay of TMS-related effects over distance, they are mostly located on gyral crowns. Depending on individual brain anatomy, we were not able to target areas with TMS that are located on the bottom of a sulcus (**a** V3a and hMT+/V5) or buried between cerebrum and cerebellum (**b** V2v, V3v, and V4)

For each coil position, we defined a point of the maximum stimulation effect on the cortical surface (see “Defining the location of TMS effects in the brain” section). Figure 4 shows a flatmap of the visual cortex (pial surface) for two participants, the defined centre points of stimulation and outlines of functional areas are superimposed. Since the effect of TMS decays as a function of distance from the coil to the cortical surface, maximum stimulation effects were often located on gyral crowns in the superior parts of the visual cortex (see Fig. 4). From this, it follows that some functional areas were targeted more often than others.

Table 1 shows the mean probability of inducing phosphenes for all stimulations that fell in respective functional areas. Stimulations of early visual cortex produced phosphenes reliably (V1 40.8 %, V2d 45.4 %, V2v 41.7 % of stimulations). Stimulation of areas along the dorsal pathway had the highest chance of inducing a phosphene (V3d 60 %, V3a 56.4 % of stimulations). At higher visual areas, stimulation seldom produced a percept (hMT+/V5 13.8 %, LOC 12.8 % of stimulations).

**Table 1:** Probability (percentage) of producing a phosphene through stimulation of functional areas

Functional area	Mean (±SEM) phosphene probability in % of stimulations	Number of stimulations	Number of participants
V1	40.8 (9.5)	260	6
V2d	45.4 (7.3)	280	7
V3d	60 (9.3)	200	7
V3a	56.4 (12.8)	120	5
V2v	41.7 (22.4)	60	3
V3v	40 (20)	20	2
V4	40 (0)	10	1
hMT+/V5	13.8 (8.9)	80	4
LOC	12.8 (5.6)	290	7

Results are averaged across participants

There were only very few stimulations that targeted ventral visual areas V3v and V4 (<50 stimulations pooled over all participants). These functional areas are hidden between the bottom part of the cerebrum and the cerebellum and are therefore hard to reach with TMS in most people (see Fig. 4). Due to the anatomical constraints, increasing the number of participants would not necessarily have resulted in a dramatic increase in the number of stimulations to these areas. We therefore present the data, but deliberately do not include these areas in the discussion of the study.

### Cortical excitability changes

TMS-related effects might build up over time during repeated stimulation, changing cortical excitability over the duration of a TMS experiment (Walsh and Pascual-Leone 2003). In this experiment, the average number of phosphene perceptions did not differ significantly over the duration of a test session: *F*(7,140) < 1, *p* = .75, and showed good test–retest reliability for all participants over eight subintervals (8 × 20 stimulations) of a test session (Chronbach’s *α* = .76). This suggests that cortical excitability did not change as a function of time or number of applied stimulations during the experiment. Given that we applied single pulse stimulation and kept a minimum stimulus onset asynchrony of 3 s, we did not expect any long-lasting effects induced by TMS.

Another concern was that cortical excitability might fluctuate for different days and therefore affect our findings at different testing sessions (Walsh and Pascual-Leone 2003). We found that the average number of phosphenes perceived in different testing session was reliable (3 test sessions; Cronbach’s *α* = .69) for all participants. This suggests that any changes in cortical excitability across testing days are unlikely to have had a strong influence on our findings.

### Location of TMS effects in the brain

To validate localization of stimulation effects with a projection method, we compared the outcome to a realistic current model for 17 coil positions (see “Defining the location of TMS effects in the brain” section). We found a good correspondence between the target areas predicted by the projection and the target areas indicated by the current model: For 14/17 coil positions, there was an exact match between the outcome of the projection method and the outcome of the current model, while for 2/17 positions the projections fell on a neighbouring area that received the second strongest stimulation. For one position, the projection method predicted stimulation of a functional area where no electric field was induced.

### Phosphene frequency with rTMS stimulation

Six participants who did not perceive phosphenes through single pulse stimulation were retested with a more powerful rTMS protocol (see “Phosphene induction with different stimulation protocols” section). Three out of six participants reported phosphenes through rTMS. Subsequently, they were able to perceive a stable percept through a single pulse phosphene screening (see “Phosphene screening” section). Two participants very sporadically reported a percept through rTMS. They did not report any percept through subsequent single pulse stimulation. One participant never reported a phosphene after rTMS or single pulse stimulation.

## Discussion

In this study, we systematically map out where in the visual cortex TMS can induce phosphenes (Fig. 2). Stimulation of the early visual cortex (V1, V2d, and V2v) and structures along the dorsal pathway (V3d, V3a) induce phosphenes reliably (see Table 1). This suggests that in these areas of the visual cortex, TMS stimulation reliably induces neural activation that will propagate to a degree at which it creates a conscious percept. These findings suggest that we can use TMS in the early and dorsal visual cortex to make causal inferences regarding the functional role of underlying areas in the human cortex.

### Probability of inducing phosphenes in the visual cortex

The probability of producing a phosphene with TMS is variable for different parts of the visual cortex. Moving the stimulation site closer to the interhemispheric cleft increases the probability of inducing a phosphene (Fig. 3). Similar findings were reported previously (Marg and Rudiak 1994; Kammer et al. 2005).

One possible explanation for this could be that the part of the cortex next to the cortical midline lies close to the scalp (Stokes et al. 2005) and should therefore receive stronger TMS-related effects (Kammer et al. 2005; Wagner et al. 2009; Stokes et al. 2013). In this study, we corrected stimulation intensity for the underlying distance between the coil and the cortical surface. We found that, after correction, stimulation intensity or distance from the coil to the underlying cortical surface did not significantly predict whether TMS would yield a percept. This suggests that we successfully controlled for these predictors of TMS efficacy.

It is also possible that only TMS-related activation of a specific neural structure or network close to the cortical midline will produce phosphenes. Different parts of the visual cortex are suggested as potential generator structures for phosphenes: the striate cortex (V1), extrastriate areas (V2/V3), cortico-cortical tracts projecting from V2/V3 back to V1 or the optic radiations as a subcortical structure (Kammer et al. 2005). Pascual-Leone and Walsh (2001) showed that phosphene perception induced through extrastriate stimulation can be disrupted through subsequent stimulation of V1. Studies of brain-lesioned patients showed that an intact V1 is necessary for phosphene perception (Cowey and Walsh 2000; Gothe et al. 2002). These findings suggest that the spread of TMS-related neural activation at the target site through a network connected to early visual structures might underlie phosphene perception. Structural connectivity between V1 and V3d has been demonstrated in non-human primates (Felleman and Van Essen 1991; Markov et al. 2014; Arcaro and Kastner 2015). In humans, strong functional connectivity between V1 and V3d during resting state fMRI suggests a similar anatomy (Heinzle et al. 2011; Genc et al. 2016). The higher susceptibility to phosphene induction that we found for dorsal areas V3d and V3a might therefore be explained by the connectivity between these areas and the early visual cortex.

Finally, intracranial parameters that we cannot control (e.g. local orientation of neurons relative to the induced current orientation) might play a key role for stimulation in the visual cortex (Wagner et al. 2009). Structures close to the midline such as the tracts projecting from V2/V3 back to V1 and the optic radiations are more prone to TMS due to their bending structure (Kammer et al. 2005). Also, with induced currents running lateral to medial, a higher number of phosphenes close to the interhemispheric cleft could be due to current orientation running perpendicular to the stimulated gyrus which marks the onset of the interhemispheric cleft (Kammer et al. 2007). However, in this study, we only found slight changes in the stimulation outcome for different current directions (see “Effects of current direction on phosphene induction” section) which makes it unlikely that the observed results are driven by an interaction between current orientation and intracranial parameters such as the orientation of local neurons to the induced current.

It is worth noting that the phosphene probabilities provided in Table 1 are specific to the left hemisphere. Previous work indicated that phosphenes can be induced in both hemispheres (Marg and Rudiak 1994; Kammer 1999; Kammer et al. 2005), and suggests that cortical excitability does not differ between hemispheres in occipital areas V2 and V3 (Kammer et al. 2001a). This gives us no reason to expect any interhemispheric differences.

### Phosphene induction in different functional areas

Our results suggest that phosphenes are induced through TMS-related neural activation in visual areas close to the midline. In this study, we predicted a location of the maximum induced current to describe TMS-related effects relative to functional areas (Okamoto and Dan 2005). We found that stimulation of early visual areas produces phosphenes reliably (V1 40.8 %, V2d 45.4 %, V2v 41.7 % of stimulations) as previously reported (Abrahamyan et al. 2011; Salminen-Vaparanta et al. 2014). However, our results suggest that TMS induces a percept most frequently when aimed at dorsal visual areas (V3d 60 %, V3a 56.4 % of stimulations).

It is conceivable that intrinsic parameters of the targeted areas might explain these results: Stimulation of neurons with larger receptive fields in V3a might produce larger phosphenes compared to neurons in V1. This could cause participants to spot phosphenes more easily after dorsal stimulation and potentially explain different susceptibility to phosphene perception at different functional areas. However, in a previous study, participants reported slightly smaller phosphenes for stimulation of the dorsal visual cortex (V3d and V3a) compared to primary visual cortex V1 (Kammer et al. 2005). In general, previous phosphene studies have reported that the overall appearance of phosphenes does not change significantly when different areas of the brain are stimulated (Kammer et al. 2005; Salminen-Vaparanta et al. 2014). This makes it unlikely that phosphene appearance systematically affected the stimulation outcome of this experiment.

The area with the highest average phosphene incidence (V3d) was the same area where individual cortical excitability was defined. One concern is that the experiment was therefore in some way biased towards ideal stimulation parameters for V3d and neighbouring areas. While we cannot rule out this possibility, we think it is unlikely to have played a major role. This is because for all different areas in the visual cortex, we controlled all stimulation parameters that we can influence extracranially (stimulation intensity, current direction, and stimulation accuracy) to induce comparable stimulation effects.

For areas in the ventral visual cortex (V3v, V4), we were only able to apply a relatively low number of stimulations in two participants (see Table 1). Due to their hidden location at the inferior ventral side of the brain, these areas are hard to reach with TMS in most participants (see Fig. 4). We therefore cannot draw any firm conclusions regarding the excitability of these areas.

In higher visual areas, TMS stimulation had a low chance of producing a phosphene (hMT+/V5 13.8 %, LOC 12.8 % of stimulations). For area hMT+/V5, these findings are unexpected as previous studies were able to induce moving phosphenes through stimulation at this area (Antal et al. 2004; Najib et al. 2010). One possible explanation could be state dependency of phosphene behaviour: The absence of any motion priming might have made it harder to spot moving phosphenes (Guzman-Lopez et al. 2011).

It is important to note here that the absence of phosphenes in some parts of the visual cortex cannot be used as an indicator that TMS did not induce neural activation. First, there is no reason to believe that one part of the brain is excitable and another not (Walsh and Pascual-Leone 2003). Second, the underlying process that is triggered by TMS and leads to phosphene perception is not understood (Kammer et al. 2005). Finally, TMS-related activation of neurons might have been below a critical threshold to induce a phosphene; however, a population of neurons was still activated (Wagner et al. 2009; Silvanto 2013). In particular, Ramos-Estebanez et al. (2007) showed that subthreshold stimulation causes substantial neural activation while no percept occurs. With the paradigm used in this experiment, we are not able to draw any conclusion from stimulations that did not yield a percept; however, it is unlikely that no neural excitation was triggered through TMS.

### Locating the effects of TMS

A key limitation of TMS studies is the unknown location and spatial specificity of TMS-related neural activation. In this study, TMS effects where assigned to a single point on the cerebral surface where the induced current is estimated to be maximal. This is based on the assumption that the impact of TMS on neural tissue is maximally initiated where currents are maximal under the centre of the coil (Wagner et al. 2009). This approach has two limitations:

First, depending on the stimulator output, the induced electric field is reported to spread approximately 100–200 mm^2^ on the cerebral surface (Wagner et al. 2009). Due to this coarse focality and individual differences in functional brain architecture, it is often not possible to constrain the induced electric field to a single functional area (Salminen-Vaparanta et al. 2012). It is possible that stimulations in this study might have activated neurons in multiple neighbouring areas.

One possibility is to define the neural activation based on a model of the theoretical current spread of TMS in the brain (Salminen-Vaparanta et al. 2014). However, this approach has its own limitations: It is currently unclear in what way the interactions between the electric current and brain tissue trigger neural firing and whether there is a linear relationship between current intensity and neural activation (Bestmann et al. 2015).

The second limitation of this approach is that neural activation due to TMS is predicted to be maximal where the induced current was maximal. However, this is not necessarily true: Recent studies have proposed that a subcomponent of the electric field that is induced by TMS can best predict stimulation outcomes in the motor cortex (Laakso et al. 2014; Janssen et al. 2015). This subcomponent is perpendicular to and directed into the cortical surface and, for TMS, maximally affects neurons situated in the sulcal wall. Most importantly, this component is not necessarily located at the electric field maximum (Laakso et al. 2014).

In this study, we used a realistic electric field model to validate the assignment of stimulation effects to functional areas based on a projection method. We found a good correspondence between the target areas predicted by the projection and the target areas indicated by the current model. This suggests that the projection method used in this study successfully identified functional areas in the visual cortex targeted by stimulation, notwithstanding the complications of localizing the effects of TMS pulses in the brain.

### Reliability of phosphenes as a signature of stimulation

A challenge in understanding the efficacy of TMS through phosphenes is to draw general conclusions based on a limited subsample. For this experiment, we recruited 30 participants but found that only 12 participants (40 %) perceived phosphenes reliably. In particular, nine participants (30 %) did not report phosphenes after any TMS stimulation. While we sought to provide the optimal conditions for phosphene perception (see “Methods” section), there are two main reasons why this might have occurred. First, it is possible they were overly conservative in their responses and were not sufficiently confident in their perception of very briefly induced phosphenes. Second, it is possible that the single pulse stimulation induced only subthreshold neural activation that was insufficient to induce a conscious percept (Wagner et al. 2009; Silvanto 2013).

To test these two possible explanations, we retested six participants who reported no percept with a more powerful rTMS protocol that was reported to induce a percept in every participant (Ray et al. 1998; Boroojerdi et al. 2002). Additionally, this protocol creates a more vivid, easy-to-spot percept (Marg and Rudiak 1994; Kammer et al. 2005).

Three participants reported phosphenes through rTMS. Notably, they also were able to perceive a stable percept through subsequent single pulse stimulation. This suggests that they had not previously spotted the percept through single pulse TMS. Two participants very sporadically reported a percept through rTMS. They did not report any percept through single pulse stimulation. One participant never reported a phosphene after rTMS or single pulse stimulation. It is possible that TMS only induced subthreshold neural activation in these participants.

In this project, we only applied single pulse stimulation while some participants were not able to gain a percept from this protocol. We did so because rTMS has certain drawbacks: The area of the induced current will be larger making it hard to locate TMS-related effects (Robertson et al. 2003). Also, repeated stimulation protocols are more prone to not only trigger action potentials during the pulse but also alter the level of neural excitability of targeted tissue over time (Wagner et al. 2009).

Finally, there is some evidence suggesting that there might be functional differences in visual neural networks between participants who report phosphenes through TMS and participants who do not. Specifically, fMRI activation has been found to differ during visual checkerboard stimulation (Meister et al. 2003) as well as TMS (Caparelli et al. 2010) for participants that do not report phosphenes. However, Caparelli et al. (2010) observed TMS-related blood oxygenation-level-dependent signal changes in both types of participants. These findings suggest that, while the behavioural outcome varies between participants, TMS does affect neural activity in both types of participants.

### Effects of current direction on phosphene induction

Recent studies which used current modelling have shown that the direction of the induced current relative to local brain anatomy has an impact on the strength of the induced electric field (Opitz et al. 2013; Laakso et al. 2014; Janssen et al. 2015). These findings are in line with a direct relationship between a change in current direction and the amount of triggered neural activation in the motor cortex (Brasil-Neto et al. 1992; Mills et al. 1992; Kammer et al. 2001b).

In the visual cortex, this relationship is less clear cut. Kammer et al. (2007) reported that individual brain regions have an ideal current direction for phosphene induction (perpendicular to the underlying gyral crown); however, these effects were marginal. We therefore chose to standardize the current direction for all coil locations. However, a standardized current direction is unlikely to stimulate a maximum number of neurons at any given location in the visual cortex. This might compromise our interpretation that a difference in susceptibility to phosphene induction was due to the intrinsic properties of different areas in the visual cortex.

We therefore conducted a control study with three participants (these were the only ones from the original participants who were available) to test whether the current direction would affect the systematic differences in susceptibility to phosphene induction that we observed. We found very slight differences for phosphene induction with different current directions as previously reported by Kammer et al. (2007). Importantly, the pattern of high, medium, or low phosphene susceptibility for different coil locations was preserved irrespective of the coil orientation. This suggests that it is unlikely that our results are specific to the lateral-to-medial current direction used during phosphene mapping.

### Effects of stimulation intensity corrections

In this study, we corrected stimulator output to induce comparable stimulation effects at different sites in the brain. However, for one participant with a high excitation threshold, all stimulations were delivered at the maximum stimulator output used in this study; hence, stimulator output was not controlled as a potential predictor of the stimulation outcome. One concern was that this might have systematically affected the overall results. However, Fig. 2a shows that stimulation results for this participant were similar to the results of most of the other participants. This shows that the overall results of this study were not systematically affected by a partial lack of stimulator output correction.

## Conclusion

Our results show that single pulse TMS can reliably induce phosphenes in early and dorsal areas of visual cortex close to the interhemispheric cleft. We propose that TMS-related maximum induced currents located at functional areas V1, V2d, V2v, V3d, and V3a can trigger a critical amount of neural activation that will propagate and create a conscious percept. Stimulation in dorsal visual areas (V3d, V3a) was most likely to induce a phosphene. This could indicate that TMS-induced extrastriate neural activation that propagates back to primary visual cortex will create phosphenes.
